# Mutation in *Staphylococcus aureus* that supports gain of function in susceptibility both to hypochlorous acid and to human neutrophils

**DOI:** 10.1093/jleuko/qiaf057

**Published:** 2025-05-28

**Authors:** Athmane Teghanemt, Katrin Schilcher, Jeffery S Kavanaugh, Alexander R Horswill, William M Nauseef

**Affiliations:** Inflammation Program and Department of Medicine, Roy J. and Lucille A. Carver College of Medicine, University of Iowa, 523 Eckstein Medical Research Building, 431 Newton Road, Iowa City, IA 52242, United States; Department of Immunology and Microbiology, School of Medicine, University of Colorado Anschutz Medical Campus, 12800 East 19th Avenue/Mail Stop 8333, Aurora, CO 80045, United States; Department of Genetics, Genomics and Cancer Sciences, University of Leicester, Adrian Building, University Road, Leicester LE1 7RH, United Kingdom; Department of Immunology and Microbiology, School of Medicine, University of Colorado Anschutz Medical Campus, 12800 East 19th Avenue/Mail Stop 8333, Aurora, CO 80045, United States; Department of Immunology and Microbiology, School of Medicine, University of Colorado Anschutz Medical Campus, 12800 East 19th Avenue/Mail Stop 8333, Aurora, CO 80045, United States; Department of Veterans Affairs Eastern Colorado Health Care System, 1700 N Wheeling St., Aurora, CO 80045, United States; Inflammation Program and Department of Medicine, Roy J. and Lucille A. Carver College of Medicine, University of Iowa, 523 Eckstein Medical Research Building, 431 Newton Road, Iowa City, IA 52242, United States

**Keywords:** antimicrobial activity, human neutrophils, hypochlorous acid, *Staphylococcus aureus*

## Abstract

Optimal antimicrobial action of human neutrophils (polymorphonuclear leukocytes [PMNs]) relies on the synergy of oxidants and granule proteins, most notably that between the granule protein myeloperoxidase (MPO) and hydrogen peroxide (H_2_O_2_) to oxidize chloride anion to produce the potent microbicide, hypochlorous acid (HOCl). However, despite the potency of HOCl, some ingested *Staphylococcus aureus* cells survive within PMNs and contribute to disease. To identify factors that support the resistance of ingested staphylococci to PMN-oxidative killing, we screened the Nebraska Transposon Mutant Library in the USA300 methicillin-resistant *S. aureus* strain for mutants that were more sensitive or resistant to HOCl. We identified a mutant in *mazF* that survived challenge with reagent HOCl better than did the parental strain. In addition, the mutant resisted killing by human PMNs, suggesting that MazF contributes to the susceptibility of *S. aureus* to HOCl-mediated damage, the ability of *S. aureus* to recover from HOCl attack, or both. To confirm the genetic basis of the MazF phenotypes, we transformed the mutant with an expression plasmid carrying the wild-type *mazF* gene or the empty vector control to complement the phenotype. The deletion mutant with the empty vector survived better in reagent HOCl and in PMNs than did the parental strain or the complemented deletion mutant. Taken together, these data suggest that in the absence of *mazF* expression, USA300 methicillin-resistant *S. aureus* better resisted, repaired, or both resisted and repaired the sublethal damage produced by HOCl alone or by antimicrobial elements in human PMNs.

## Teaser

Mutation of *mazF* in *Staphylococcus aureus* decreases susceptibility to killing by hypochlorous acid and by human neutrophils.

## 1. Introduction

Human polymorphonuclear leukocytes (PMNs) dominate the initial innate immune cellular response to invading microorganisms such as *Staphylococcus aureus.*^1,2^ After phagocytosis of an organism, PMNs sequester their prey in membrane-bound phagosomes, wherein antimicrobial agents create a toxic environment that promotes death and degradation of the ingested microbe. Optimal PMN microbicidal action relies on collaboration between oxidants generated by the phagocyte NADPH oxidase and an array of proteins stored in PMN granules. Among phagocytes, PMNs are unique, as they possess myeloperoxidase (MPO) in their granules and thus have the singular capacity to oxidize chloride and thereby generate hypochlorous acid (HOCl), a potent microbicide. We and others have demonstrated that HOCl is generated in the phagosomes of human PMNs and mediates chlorination of bacterial proteins that correlates with death of the organism.^3–5^ The frequent and often fatal staphylococcal infections incurred by patients with chronic granulomatous disease, an inherited disorder associated with the absence of a functional phagocyte NADPH oxidase,^6^ along with the defective killing of *S. aureus* by MPO-deficient PMNs in vitro,^7^ and reviewed in Klebanoff et al.,^8^ underscore the importance of oxidant generation and HOCl production for optimal antistaphylococcal activity in human PMNs.

Despite robust oxidant-dependent human PMN antimicrobial activity, approximately 20% to 30% of the ingested *S. aureus* inoculum resists killing and remains viable within PMNs.^5,9^ Viable *S. aureus* within PMNs can transfer infection to naïve animals in murine models, support metastatic infection in humans, and promote lysis of PMNs that amplifies local tissue inflammation.^9–11^ Furthermore, “persisters,” the nongrowing dormant bacteria that display high-level antibiotic tolerance and survive in phagocytes and biofilms,^12^ may contribute to the chronic and relapsing nature of staphylococcal infections. We hypothesize that an inducible or provoked response of *S. aureus* within human PMN phagosomes contributes to the observed survival of *S. aureus* in PMNs and recalcitrance of *S. aureus* infections. The mechanisms underlying the capacity of some *S. aureus* to survive in PMNs are not defined but have clinical importance. To gain insight into genetic determinants that contribute to *S. aureus* responses to HOCl, we screened a transposon-mutant library in USA300 methicillin-resistant *S. aureus* and identified a mutant in *mazF* that exhibited altered susceptibility to HOCl and to PMN-mediated killing. Taken together, our data demonstrate that in the absence of *mazF*, the mutant USA300 methicillin-resistant *S. aureus* successfully resisted, repaired, or both resisted and repaired damage produced by reagent HOCl or by antimicrobial elements in the phagosome of human PMNs.

## 2. Materials and methods

All reagents were purchased from Fisher Scientific unless otherwise indicated. Heparin was purchased from Fresenius Kabi USA LLC, clinical grade dextran T500 from Pharmacosmos, and Ficoll-Hypaque PLUS from GE Healthcare. Sterile endotoxin-free water and 0.9% sterile endotoxin-free sodium chloride were obtained from Baxter (Deerfield, IL).

### 2.1 Bacterial strains and plasmids

Bacterial strains and plasmids are listed in Table 1 and primers are listed in Table 2. Genomic DNA of *S. aureus* strains was isolated using the Puregene cell kit (QIAGEN; Cat#158567), including a lysis step with lysostaphin (100 ng/μL; ABMI Products LLC; Cat#LSPN). Polymerase chain reaction (PCR) products were purified with either the QIAquick PCR Purification Kit (QIAGEN; Cat#28106) or the QIAquick Gel Extraction Kit (QIAGEN; Cat#28706). Purification of all plasmids was performed with the QIAprep Spin Miniprep Kit (QIAGEN; Cat#27106). DNA sequencing of the constructed plasmids was performed at the Molecular Biology Service Center at the University of Colorado Anschutz Medical Campus.

**Table 1.** Bacterial strains and plasmids.

	Description	Reference
DC10B	*Escherichia coli* cloning strain	^13^
JE2	*S. aureus* USA300 LAC derivative	^14^
NE1833	*S. aureus mazF*::ɸNΣ from NTML	^14^
AH5685	*S. aureus* USA300 JE2 *mazF*::ɸNΣ	This paper
AH1263	*S. aureus* USA300 CA-MRSA LAC*	^15^
AH5684	*S. aureus* USA300 LAC* *mazF*::ɸNΣ	This paper
AH5828	*S. aureus* USA300 JE2 carrying pCM29	This paper
AH5830	*S. aureus* USA300 JE2 *mazF*::ɸNΣ carrying pCM29	This paper
AH6017	*S. aureus* USA300 LAC* carrying pALC2073 (pEMPTY)	This paper
AH6018	*S. aureus* LAC* *mazF*::ɸNΣ carrying pALC2073 (pEMPTY)	This paper
AH6019	*S. aureus* USA300 LAC* *mazF*::ɸNΣ carrying pKAS110	This paper
**Plasmids**		
pALC2073	Expression vector, Amp^R^/Cm^R^	^16^
pKAS110	pALC2073_*mazF* with native RBS, Amp^R^/Cm^R^	This paper
pCM29	sGFP expression vector, Amp^R^/Cm^R^	^17^

pEMPTY, empty plasmid control; pKAS110, gene complementation; RBS, ribosome binding site.

**Table 2.** Primer list.

Primer	5′–3′ sequence	Application	Reference
YSC22	AGGATGGAATACCTAAAGCCT	*mazF* transposon confirmation	This paper
YSC23	GGGGGAGTCAGACCTGTAGT	*mazF* transposon confirmation	This paper
KAS452	ccccgagctcTAGACGCCTAATTTTTCTGGTG	Construction primer	This paper
KAS453	ccccggtaccTGGAGGCGAATGAATGATTAGACGAGGA	Construction primer	This paper
KAS88	GGTGTGAAATACCGCACAGA	Sequencing primer	This paper
KAS89	GGCGAGTTTACGGGTTGTTA	Sequencing primer	This paper

Restriction enzyme sites are underlined.

### 2.2 Construction of *mazF* transposon mutant and complementation plasmids

Bacteriophage transductions between *S. aureus* strains were performed with phage 11, as described previously.^18^ The mariner-based transposon *bursa aurealis* mutation in *mazF* (NE1833) from the Nebraska Transposon library (ΦNΣ)^14^ was confirmed by PCR with the primers YSC22/YSC23. A complementation plasmid carrying the *mazF* gene with its native ribosome binding site was constructed. The *mazF* with its native ribosome binding site was amplified with primers KAS452/KAS453. The resulting DNA fragments were digested with *Kpn*I and *Sac*I and ligated into plasmid pALC2073, which was digested with the same restriction enzymes. The resulting complementation plasmid pKAS110 was transferred into *E. coli* DC10B by electroporation, sequenced with primers KAS88 and KAS89, and transferred into *S. aureus* LAC *mazF*::ΦNΣ, as previously described.^13,19^ Empty vector controls (pALC2073) were electroporated into *S. aureus* LAC* WT and *mazF*::ΦNΣ.

### 2.3 Generation of fluorescent bacteria

Constitutive GFP-expressing plasmid pCM29^17^ was introduced to JE2 WT and *mazF*::ΦNΣ strains by bacteriophage transduction performed with phage 11, as described previously,^18^ resulting in strains AH5828 and AH5830, respectively.

### 2.4 Screening of mutants from transposon library

Screening for susceptibility to HOCl or H_2_O_2_ was performed in a previously defined minimal media (MM) without methionine (MMWM).^4^ As detailed in the Results, experiments to define how methionine, cysteine, or both would consume exogenously added HOCl were performed with MMWM, MM lacking cysteine (MMWC), or MM lacking both methionine and cysteine. Using a multichannel pipette, samples of bacteria from frozen 96-well transposon library plates were transferred to 96-well plates containing 200 μL/well of tryptic soy broth (TSB). Plates were placed in a humidified shaking incubator (Stuart microplate incubator shaker SI 505) at 37 °C and 1,000 rpm for 16 h. The following day, absorbance at 600 nm (A_600_) for each well was measured. Each well was subcultured at a starting A_600_ of 0.1 in a sterile 96-well plate containing 200 μL/well of TSB at 37 °C and 1,000 rpm for 3 h. Plates were centrifuged (3,400 rpm at 4 °C), supernatants discarded, and pellets resuspended in 100μL/well of MMWM. A_600_ was determined and three plates for each screen were created, using an initial A_600_ 0.16 in 200 μL/well of MMWM. Each set of three plates was incubated for 45 min in the humidified shaking incubator at 37 °C and 1,000 rpm, after which 10 μL of phosphate-buffered saline (PBS) was added to plate 1, 10 μL of 2 mM HOCl to plate 2, and 10 μL of 6 mM HOCl to plate 3. Plates were incubated in the humidified shaking incubator at 37 °C and 1,000 rpm for 16 h, after which time the A_600_ for each well was measured.

### 2.5 Chlorination

The amount of residual HOCl present in the media of interest after the addition of defined amounts of HOCl was quantitated as the capacity to chlorinate taurine, as described previously.^20^ Stock HOCl was diluted 1:100, and the concentration determined spectrophotometrically using the molar extinction coefficient (ε_M_) 350 M^−1^cm^−1^ at 292 nm. This stock, 1.36 M, was diluted again to generate a series of working solutions (0.33 to 33 mM). Two microliters of working stock HOCl solutions were diluted to 200 μL in PBS, TSB, or the defined MM of interest (MM or MMWM) in triplicate in a 96-well plate. Plates were incubated in a microplate shaking incubator for 10 min at 37 °C, after which 20 μL of 50 mM taurine was added to each well (final concentration 5 mM) followed by 50 μL trimethylbenzidine in acetate buffer.^20^ A_650_ was measured and HOCl concentration determined as described.^20^

### 2.6 Isolation of human PMNs

Human PMNs were isolated from venous blood collected from healthy volunteers, as previously described.^21^ Written consent was obtained from each volunteer in accordance with a protocol approved by the Institutional Review Board for Human Subjects at the University of Iowa. Briefly, PMNs were isolated from heparinized venous blood using dextran sedimentation followed by a density gradient separation on Ficoll-Hypaque PLUS. After hypotonic lysis of erythrocytes, PMNs were resuspended in Hanks' Balanced Salt Solution (HBSS) without calcium or magnesium to 2.0 × 10^7^ cells/mL. For functional assays of phagocytosis and killing, PMNs were pelleted and resuspended in HBSS with divalent cations calcium and magnesium and 1% human serum albumin.

### 2.7 Phagocytosis and killing by PMNs

Phagocytosis and killing assays were performed as described previously.^5,22^ Phagocytosis was quantitated by flow cytometry using green fluorescent protein (GFP)–expressing *S. aureus.*^5,22^ To prevent bleaching of bacteria associated GFP, DPI (diphenyleneiodonium) was added to inhibit activity of the NADPH oxidase.^23^ DPI does not alter phagocytosis of *S. aureus* by human PMNs.^5^ Mean fluorescence intensity for each sample was calculated using FlowJo (FlowJo, LLC; version 10.10) as the geometric mean of the (GFP^+^ population) × (percent of cells gated).

To quantitate PMN-mediated killing of ingested *S. aureus*, GFP-expressing bacteria opsonized with pooled human serum were mixed with PMNs at the desired multiplicity of infection (MOI) in a 5-mL round-bottom polypropylene tube and tumbled for 10 min at 37 °C. Following 10 min of phagocytosis, cells were spun at 500 *g* for 5 min and remaining extracellular bacteria were aspirated into waste. PMNs containing bacteria were then resuspended in HBSS with divalent cations containing 1% human serum albumin. At indicated time points, PMNs were removed from the tube and assayed for *S. aureus* viability. Bacterial viability was routinely determined by PMN lysis using H_2_O brought to a pH of 11 with NaOH immediately before use,^24^ subsequent plating of serial dilutions on tryptic soy agar plates overnight, and enumeration of colony-forming units.^24^

### 2.8 Statistical analysis

Statistical comparisons were performed using either one- or two-way analysis of variance followed by Turkey's post-tests. All analyses used GraphPad Prism software (GraphPad Software; version 10). *P* values <0.05 were considered statistically significant.

## 3. Results

### 3.1 Optimizing culture conditions for screening

Recognition that HOCl reacts avidly with a wide variety of biomolecules, reviewed in Davies,^25^ made the selection of the culture medium for screening of the *S. aureus* Nebraska Transposon Mutant Library (NTML) critical. Components of nutrient-rich media such as the sulfur-containing amino acids methionine and cysteine, react avidly with HOCl and thus would be significant competitive substrates when screening for susceptibility to HOCl. In fact, Ashby et al.^26^ demonstrated that 200 μM HOCl is completely quenched when added to TSB.

**Fig. 1.**
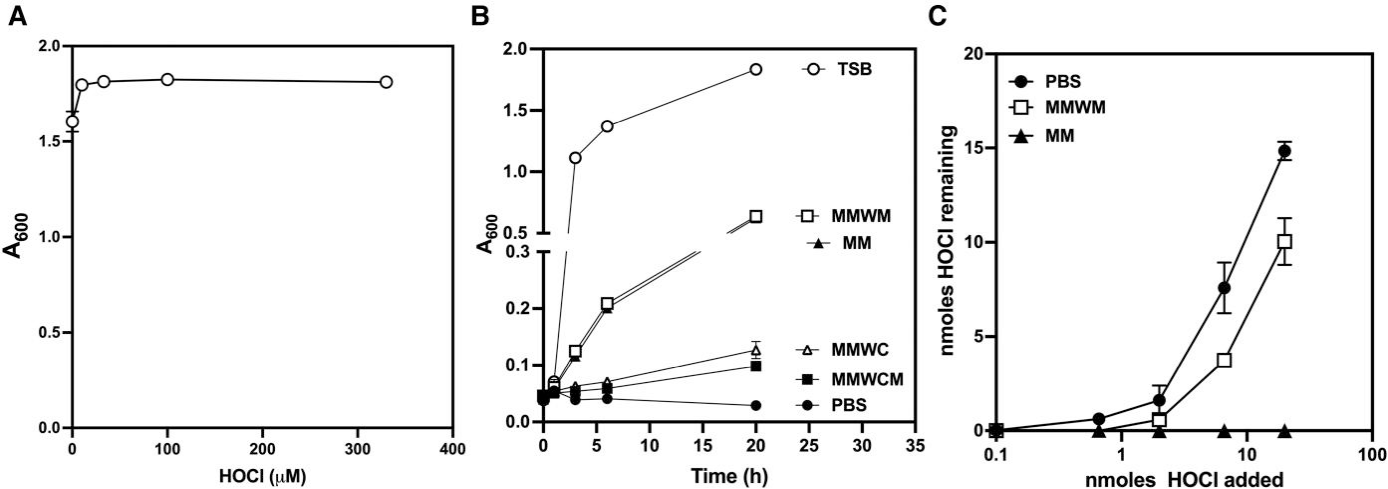
Microbicidal activity of HOCl in TSB or defined MM. (A) Viability of wild-type *Staphylococcus aureus* USA300 in TSB after the addition of HOCl (0 to 350 μM). (B) Growth of wild-type *S. aureus* USA300 in TSB, defined MM, defined MMWM, defined MMWC, MM without cysteine or methionine (MMWCM), or PBS. (C) Recovery of HOCl added to PBS, MM, or MMWM.

As expected, given the presence of reactive amino acids and proteins in TSB, the addition of HOCl arrested but failed to reduce microbial growth even at the highest amounts of HOCl added (Fig. 1A), likely a manifestation of reactions with media constituents that consumed the added HOCl. We compared growth of *S. aureus* in a defined MM previously developed in our laboratory^4^ without methionine (MMWM), without cysteine (MMWC), or without both methionine and cysteine with growth in TSB (Fig. 1B). Whereas *S. aureus* failed to grow in PBS and replicated best in TSB, growth in the defined MM varied with respect to the presence of methionine or cysteine. Compared with viability in TSB, growth was poor in defined MM that lacked cysteine (MMWC and MM without both methionine and cysteine) and intermediate in media without methionine but with cysteine (MMWM). We next compared the ability to recover added HOCl from MM with or without methionine (Fig. 1C). Nearly 80% of added HOCl was recovered from MMWM. Taken together with the ability of MMWM to support growth, these data demonstrated that MMWM would be a suitable medium to screen the transposon mutant library for relative susceptibility to added HOCl.

### 3.2 Identification of NE1833 as mutant with relative resistance to killing

**Fig. 2.**
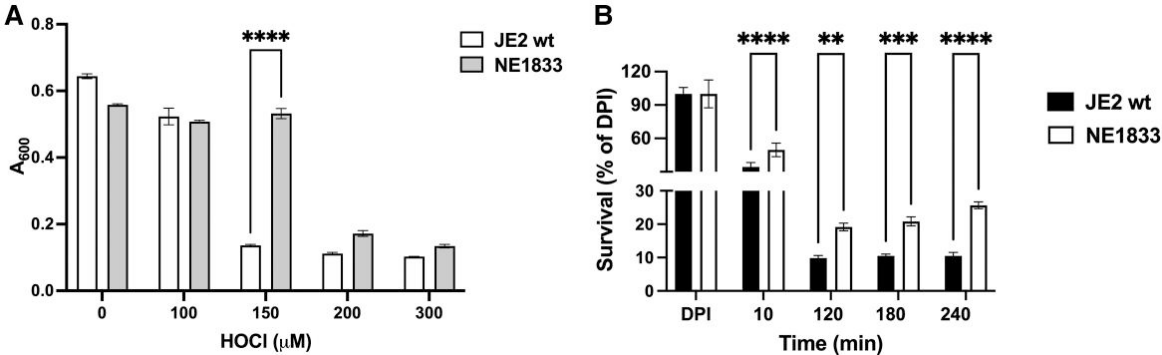
Survival of *Staphylococcus aureus* wild-type and the NTML mutant NE1833 in the presence of HOCl or after ingestion by PMNs. (A) *S. aureus* wild-type (JE2 wt) or the NTML mutant NE1833 were cultured in the presence of HOCl (0 to 300 μM) for 16 to 18 h, after which A_600_ was measured. Data shown are representative of three separate experiments, each done in quadruplicate. ****P < 0.0001. (B) Survival of JE2 wt or NE1833 fed to PMNs at MOI 1:1 from 0 to 240 min after phagocytosis. Data shown are representative of experiments using two unrelated donors, each done in sextuplicate. **P < 0.02, ***P < 0.0008, ****P < 0.0001.

Compared with growth of the parental wild-type strain of *S. aureus* (JE2 wt), one mutant (NE1833) within the screened NTML grew to a significantly higher density 16 h after exposure to 150 μM HOCl in MMWM (Fig. 2A), suggesting that, under the experimental conditions used, NE1833 was less susceptible to HOCl-mediated attack, repaired HOCl-dependent damage more readily, or both, when compared with the behavior of the parental strain. Furthermore, when exposed to continuous source of HOCl, using a defined system of MPO, H_2_O_2_, and chloride that mirrors HOCl production and oxidant-dependent antimicrobial activity in the phagosomes of human neutrophils,^3,27,28^ NE1833 demonstrated the same differential survival in comparison with the parental strain (data not shown).

To determine if the relative tolerance of NE1833 to HOCl-dependent damage influenced the fate of NE1833 in human neutrophils, we compared the survival of NE1833 after phagocytosis by neutrophils. Serum-opsonized *S. aureus*, wild-type and NE1833, were fed to human neutrophils (MOI 1:1) for 10 min, after which uningested bacteria were removed by centrifugation, and neutrophils containing ingested *S. aureus* were incubated at 37 °C for varied times (20 to 240 min). Neutrophils were lysed, and viable intracellular bacteria were enumerated. At each timepoint examined, the number of surviving *S. aureus* were significantly greater for NE1833 compared with the parental strain (Fig. 2B). Taken together, these data demonstrate that NE1833 withstood the antimicrobial action of reagent HOCl and of human neutrophils better than did the parental strain.

### 3.3 Identification of mazF as mutation in NE1833

**Fig. 3.**
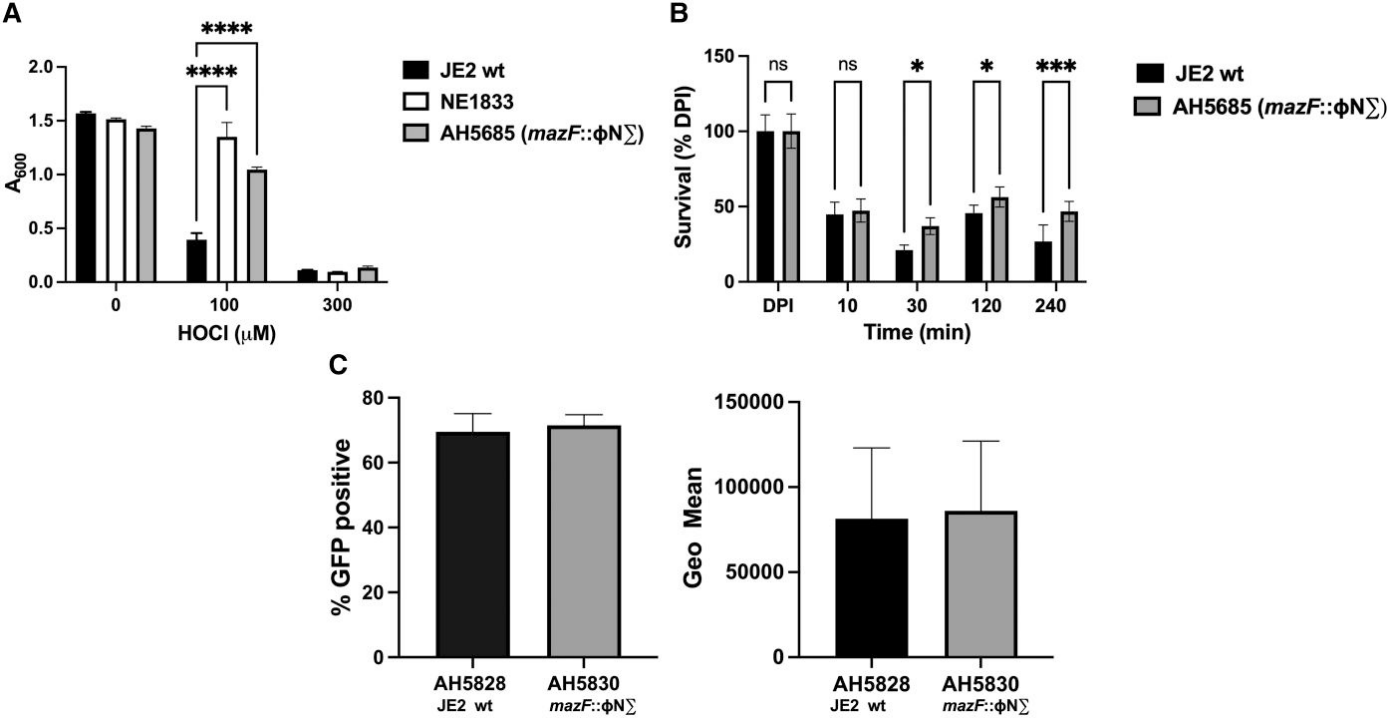
Survival of JE2 wt, NE1833, and the *mazF*::ɸN∑ mutant in the presence of HOCl or after ingestion by PMNs. (A) JE2 wt, NE1833, and the targeted *mazF* transposon mutant (*mazF*::ɸN∑, AH5685) cultured in the presence of HOCl (0 to 300 μM) for 16 to 18 h, after which A_600_ was measured. Data shown are representative of two separate experiments, each done in quadruplicate. Differences between viability of JE2 wt and either NE1833 or *mazF*::ɸN∑ (AH5685) were statistically significant (*P* < 0.0001), whereas there was no statistically significant difference between NE1833 and *mazF*::ɸN∑ (AH5685). (B) Survival of JE2 wt or *mazF*::ɸN∑ (AH5685) 10 to 240 min after phagocytosis by PMNs (MOI 1:1). Data shown are representative of experiments using four unrelated donors, each done in sextuplicate. (C) Phagocytosis of GFP-expressing parental JE2 wt (AH5828) and *mazF*::ɸN∑ (AH5830) by human neutrophils, assessed as percent GFP-positive PMNs (left) or Geometric mean of fluorescence (right). Data are mean ± SEM from three unrelated donors, each done in triplicate. There was no statistically significant difference between the two strains in their uptake by neutrophils. *P < 0.035 , ***P < 0.0002, ****P < 0.0001. ns, not significant.

Based on the annotation of the *S. aureus* NTML, we determined that the mutation associated with NE1883 is located in the *mazF* gene. However, to eliminate the possibility of secondary site mutations and to confirm that the phenotype of NE1833 resulted solely from the mutation in *mazF*, NE1833 was backcrossed into a new JE2 wt background. The resulting mutant strain, AH5685 (*mazF*::ɸN∑) (see Table 1) exhibited a significantly higher survival advantage over the parental JE2 wt strain at 100 μM HOCl, mirroring the behavior of the *mazF* transposon mutant NE1833 (Fig. 3A). Furthermore, AH5685 recapitulated the survival phenotype of NE1833 when fed to human neutrophils (Fig. 3B). Differential survival in human neutrophils was not due to differences in ingestion of bacteria, as phagocytosis of JE2 wt and *mazF*::ɸN∑ were not significantly different (Fig. 3C).

Taken together, these data demonstrate that a mutation in *mazF* resulted in better survival after challenge with reagent HOCl, an MPO-dependent HOCl-generating system, or human neutrophils.

### 3.4 Complementation of *mazF* restores the phenotype of the parental strain

The final step in linking *mazF* to the phenotype of NE1833 was to create and evaluate a complemented *mazF* mutant strain. Given that *S. aureus* USA300 JE2 is a plasmid-cured derivative of *S. aureus* USA300 LAC*, we constructed another set of strains in the original, clinically relevant USA300 LAC* background. We compared the survivals of the parental strain USA300 LAC* with pEMPTY (AH6017), the isogenic USA300 LAC* *mazF*::ΦNΣ mutant with pEMPTY (AH6018), and the isogenic USA300 LAC* *mazF*::ΦNΣ mutant with pKAS110 (AH6019), over a range of HOCl concentrations (0 to 250 μM).

**Fig. 4.**
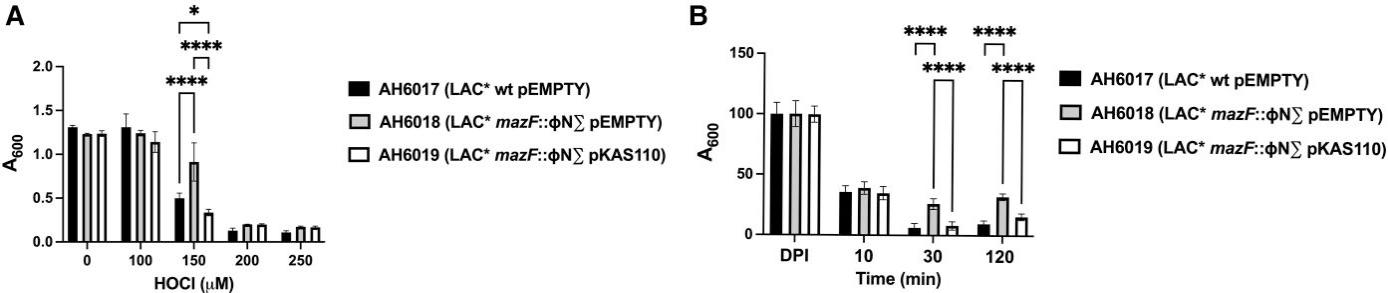
Viability of *S. aureus* LAC* wt, the *mazF*::ɸN∑ mutant and the complemented *mazF*::ɸN∑ strain in HOCl or PMNs. (A) Survival of LAC* wt (AH6017), the *mazF*::ɸN∑ mutant (AH6018), and the complemented *mazF*::ɸN∑ mutant strain (AH6019) after 16 to 18 h in HOCl (0 to 250 μM). Data shown are representative of three separate experiments, each done in quadruplicate. *P < 0.01, ****P < 0.0001. (B) Survival of LAC* wt (AH6017), the *mazF*::ɸN∑ mutant (AH6018), and the complemented *mazF*::ɸN∑ mutant strain (AH6019) 10, 30, or 120 min after ingestion by PMNs (MOI 2.5:1). Data shown are representative of experiments using five unrelated donors, each done in sextuplicate (*P* < 0.0001). pEMPTY, empty plasmid control; pKAS110, *mazF* gene complementation.

Differential survival in the presence of 150 μM HOCl was observed, with the mutant surviving significantly better than did either the parental strain or the complement (Fig. 4A). The same was true when survival of the 3 strains in human neutrophils was compared. Thirty minutes after phagocytosis by human neutrophils, survival of the *mazF* mutant was significantly greater than that of the parental strain or of the complement (Fig. 4B). The difference was not inoculum-dependent, as the same differential survival was seen at MOI 5:1 (data not shown). Restoration of the wild-type phenotype in the complemented mutant strain confirmed that mutation of *mazF* was responsible for the observed better survival of the mutant when challenged with HOCl or after ingestion by human neutrophils.

### 3.5 Effect of *mazF* on recovery from HOCl-mediated sublethal damage

**Fig. 5.**
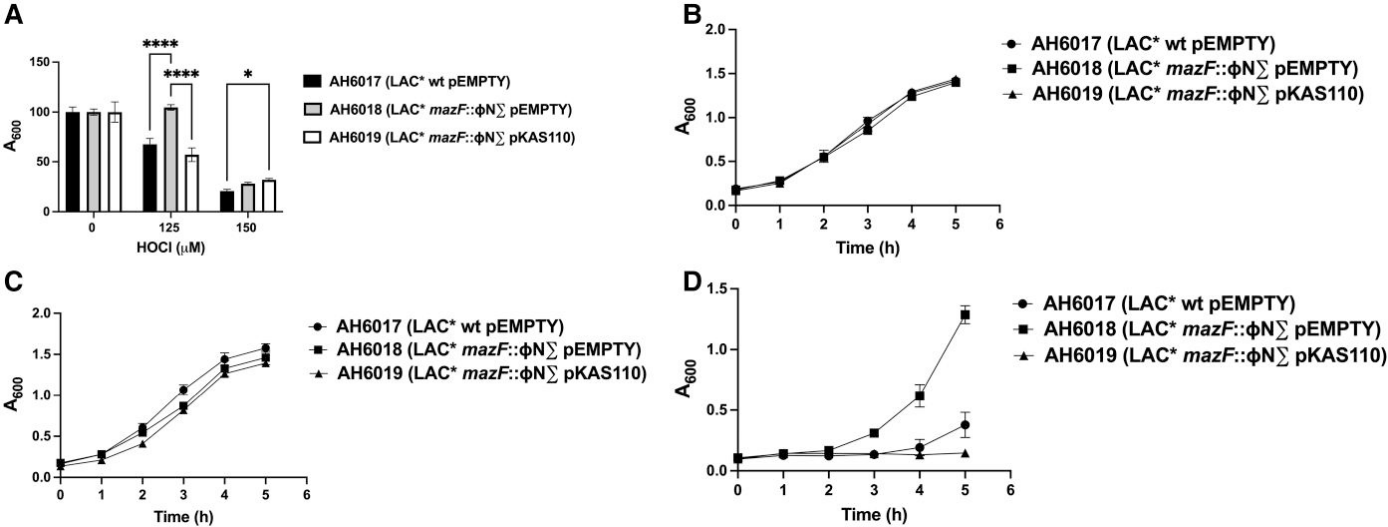
Recovery of *S. aureus* LAC* wt, the *mazF*::ɸN∑ mutant and the complemented *mazF*::ɸN∑ strain after exposure to HOCl. (A) Survival of *S. aureus* LAC* wt (AH6017), the *mazF*::ɸN∑ mutant (AH6018), and the complemented *mazF*::ɸN∑ mutant strain (AH6019) after 16 to 18 h in 0, 125 μM, or 150 μM HOCl (n = 3, each done in quadruplicate). (B) Growth over 5 h in TSB of LAC* wt (AH6017), the *mazF*::ɸN∑ mutant (AH6018), and the complemented *mazF*::ɸN∑ mutant strain (AH6019) recovered after 16 to 18 h in MMWM without HOCl (n = 3, each done in quadruplicate). (C) Growth over 5 h in TSB of LAC* wt (AH6017), the *mazF*::ɸN∑ mutant (AH6018), and the complemented *mazF*::ɸN∑ mutant strain (AH6019) recovered after 16 to 18 h in MMWM with 125 μM HOCl (n = 3, each done in quadruplicate). (D) Growth over 5 h in TSB of LAC* wt (AH6017), the *mazF*::ɸN∑ mutant (AH6018), and the complemented *mazF*::ɸN∑ mutant strain (AH6019) recovered after 16 to 18 h in MMWM with 150 μM HOCl (n = 3, each done in quadruplicate). pEMPTY, empty plasmid control; pKAS110, *mazF* gene complementation. *P < 0.01, ****P < 0.0001.

The enhanced survival of the *mazF* mutant to HOCl-mediated damage and human neutrophil antimicrobial action may reflect a greater inherent resistance to attack, an enhanced capacity to repair and recover from sublethal damage, or both. To explore the possibility that more robust repair contributed to the observed phenotype, we challenged the three strains of *S. aureus*, namely parental, mutant, and complemented mutant, to a range of concentrations of HOCl (0 to 150 μM) in MMWM. After 18 h at 37 °C, the A_600_ for each was measured (Fig. 5A). As expected given the identical growth curves for all three strains, growth was the same in the absence of added HOCl. Consistent with these data, the A_600_ for the culture of the *mazF* mutant at 125 μM HOCl was significantly greater than that of either the parental strain or the complemented strain (Fig. 5A). At 150 μM HOCl, growth in all three strains was inhibited.

Surviving bacteria at 0, 125 μM, and 150 μM HOCl were pelleted, resuspended at the same density in TSB, and cultured for 6 h, with A_600_ measured each hour. All three strains recovered from 0 or 125 μM HOCl grew to a similar extent when resuspended in TSB (Fig. 5B and C, respectively). In contrast, resuspension in TSB of the *mazF* mutant recovered from 150 μM HOCl grew significantly better than did the parental strain or the complement (Fig. 5D), suggesting that the capacity of the *mazF* mutant to repair and recover from HOCl-mediated damage contributed to its better survival.

### 3.6 Effect of *mazF* on susceptibility to H_2_O_2_ and recovery from H_2_O_2_-mediated sublethal damage

**Fig. 6.**
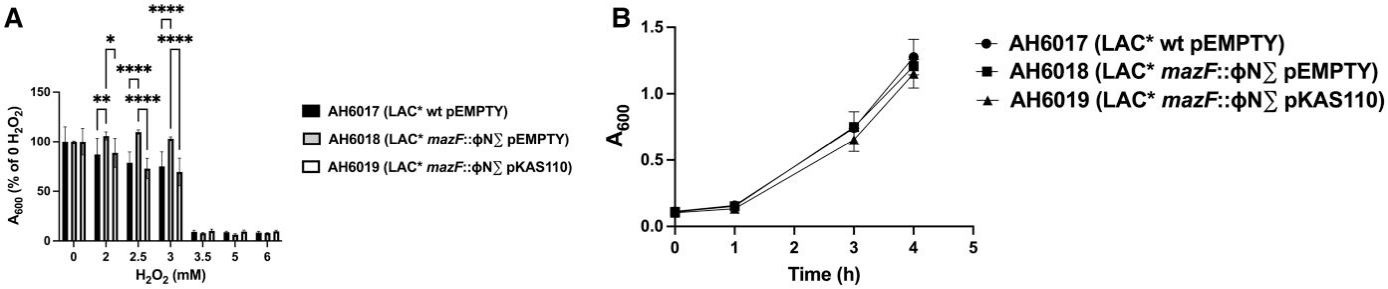
Differential recovery of *S. aureus* LAC* wt, the *mazF*::ɸN∑ mutant and the complemented *mazF*::ɸN∑ strain after exposure to HOCl or H_2_O_2_. (A) Survival of *S. aureus* LAC* wt, the *mazF*::ɸN∑ mutant and the complemented *mazF*::ɸN∑ strain after 16 to 18 h in H_2_O_2_ (0 to 6 mM). (B) Isolates recovered from sublethal damage at 3 mM H_2_O_2_ were pelleted and resuspended in TSB and cultured for 0 to 4 h. pEMPTY, empty plasmid control; pKAS110, *mazF* gene complementation. *P < 0.01, **P < 0.0079, ****P < 0.0001.

Whereas exposure of bacteria to HOCl is restricted to the lumen of the neutrophil phagosome, oxidant stress from H_2_O_2_, endogenously from microbial metabolism or exogenously from the environment, is a constant challenge for microbes.^29^ To determine if the deletion of *mazF* influenced also the susceptibility of *S. aureus* to H_2_O_2_, we compared viability of *S. aureus* over a range of H_2_O_2_ concentrations in the same culture medium and experimental conditions as used for HOCl challenges. In general, the *mazF* mutant exhibited better survival than did parental or complemented strains at 3.73 ± 0.41 mM (n = 9) (Fig. 6A), a discriminating concentration nearly 30-fold higher than that for differential susceptibility of the *mazF* mutant to HOCl (Fig. 5A).

Given our observation that the *mazF* mutant recovered from sublethal damage from HOCl compared with the parental strain or the complement (Fig. 5D), we assessed the capacity of the *S. aureus* strains to recover from sublethal damage mediated by H_2_O_2_. Growth of all three strains was suppressed by exposure to 3.5 mM H_2_O_2_ (Fig. 6A) under the same experimental conditions as in Fig. 5A. In contrast to the greater recovery of the *mazF* mutant to 150 μM HOCl (Fig. 5D), the *mazF* mutant did not recover from attack by 3.5 mM (Fig. 6B). Taken together, the differential microbial susceptibilities and responses to the two oxidants, HOCl and H_2_O_2_, suggest different underlying mechanisms.

## 4. Discussion

As the circulating blood cells that react early and rapidly to invading microbes, human neutrophils produce and employ multiple antimicrobial agents to respond to the remarkably diverse presentations by potential pathogens (reviewed in DeLeo and Nauseef.^6^) For intraphagosomal killing of bacteria such as *S. aureus*, efficient oxidant-dependent responses rely on the production of HOCl by the synergistic interaction of the neutrophil granule protein MPO and H_2_O_2_ to oxidize chloride anions and promote the early phase killing in neutrophils,^3^ with alpha defensins and other granule proteins mediating later events.^6^ Without oxidants from a functional NADPH oxidase, as occurs in patients with chronic granulomatous disease, or MPO, as in hereditary MPO deficiency, the killing of ingested *S. aureus* is absent or profoundly retarded, respectively.^7^ Although efficient and potent, staphylocidal activity in human neutrophils is incomplete, and a substantial fraction of ingested *S. aureus* survive within phagosomes after ingestion by human neutrophils ex vivo.^1,2,9,11,30^ Reasoning that the ability of ingested *S. aureus* to survive in the presence of reagent HOCl and in phagosomes of human neutrophils reflects in part a shortcoming of HOCl-dependent attack, we screened the *S. aureus* NTML for mutants that exhibited altered susceptibility to HOCl- and neutrophil-mediated killing. Unexpectedly, we identified a *mazF* mutant that demonstrated significantly greater survival against HOCl compared with that of the parental strains. MazF is a toxin with endoribonuclease activity and part of the well-studied MazEF chromosomal toxin-antitoxin system, which has been implicated in biofilm formation, persistence, and antibiotic resistance.^31,32^

Bacterial toxin-antitoxin systems are known to play critical roles in environmental stress response, as they are closely integrated with other regulatory networks.^33^ The *mazEF* operon is located directly upstream of the *rsbUVWsigB* cluster, which encodes the SigB locus. SigB serves as a key regulator of the response of *S. aureus* to H_2_O_2_ and HOCl-mediated stress.^34,35^ Transcription from different promoters results in three distinct transcripts: one for *mazEF*, one for *rsbUVWsigB*, and one spanning the entire *mazEF-rsbUVWsigB* region.^36^ Our study employs transposon mutants in *mazF* that could potentially disrupt the downstream *rsbUVWsigB* transcriptional unit. However, when the transposon mutants were complemented by expressing *mazF* from a plasmid, the restoration of the phenotype in our complemented strain ruled out the possibility that the observed *mazF* mutant phenotype was due to a polar effect of the transposon mutation on the *mazEF-rsbUVWsigB* transcriptional unit. Thus, the observed phenotype is likely linked directly to the endoribonuclease activity of MazF. The MazF endonuclease specifically cleaves RNA at UACNUA sequence motifs that are found within the *rsbUVWsigB* operon leading to decreased messenger RNA levels of *rsbV*, *rsbW*, and *sigB* transcripts upon *mazF* overexpression.^37^ This suggests that the observed phenotypes in this study could be directly related to the reduced cleavage of *rsbUVWsigB* mRNA in *mazF* mutant strains, in which the absence of MazF-meditated cleavage may lead to elevated levels of these transcripts, potentially enhancing the SigB-regulated stress response and augmenting the ability of the cell to cope with oxidative stressors such as H_2_O_2_ and HOCl.

Our data demonstrate that the *mazF* mutant was resistant to HOCl and to killing by normal human neutrophils. We do not (and cannot) extrapolate from the resistance of the mutant to HOCl-dependent killing to conclude that the survival of the mutant in neutrophils was a manifestation of its resistance to HOCl alone. If it were the case that elevated resistance to HOCl were the sole basis for increased survival of the *mazF* mutant, one might predict that the mutant would have no survival advantage in neutrophils in which MPO were absent or inhibited pharmacologically. Currently, it is very challenging to explore this possibility experimentally. To our knowledge, an effective and irreversible MPO inhibitor that penetrates neutrophil granules (akin to the action of diisopropylfluorophosphate inhibition on serine proteases in human neutrophil granules)^38^ is lacking, and our generous MPO-deficient blood donors are no longer available to us. Beyond the technical difficulties, such a prediction would fail to recognize that microbicidal activity in human phagosomes is complex, multifactorial, and in many ways an emergent phenomenon, wherein the sum of the parts is not more than each part summed but rather is different from each part summed. Eliminating MPO from the phagosome would change the composition of reactive agents available to interact with each other as well as with target bacteria, resulting in a system likely very different from one in which MPO is present. In the absence of MPO, more H_2_O_2_ would accumulate in the phagosome, as much of the H_2_O_2_ in normal neutrophils is rapidly consumed by reaction with MPO to generate HOCl. Accordingly, MPO-deficient neutrophils release more H_2_O_2_^39^ and more enzymatically active proteases extracellularly and into their phagosomes than do normal neutrophils, as the MPO-dependent oxidants inactivate elastase, proteinase 3, cathepsin G, lysozyme, and vitamin B12 binding protein as well as metalloproteinases and α1-antitrypsin.^40–43^ Of note, these same phenomenon, namely the absence of oxidant-dependent inactivation of granule enzymes, pertains to neutrophils from patients with chronic granulomatous disease, in which the oxidant component needed to support HOCl production is missing (see Voetman et al.^40^ and footnote 2 on page 1547 discussing MPO-deficient neutrophils). The complexity of these multiple overlapping and interacting biochemical events undermines efforts to determine precisely the mechanisms neutrophils use to kill ingested bacteria. Given this complex and interacting biochemistry, even if the *mazF* mutant shows no phenotype when challenged by neutrophils lacking MPO activity, it would not be possible to conclude with certainty that increased survival of the mutant was due solely to increased resistance to HOCl.

Understanding of the interplay between *S. aureus* and elements of the human immune system continues to evolve and underscores the reciprocal influences of host and microbe (reviewed in Howden et al.^44^) Our findings demonstrate that ingested *S. aureus* detect and react to the antimicrobial agents generated in the human neutrophil phagosome and highlight the dynamic and complex interactions that contribute to phagocyte-dependent host defenses.
